# Movement Patterns in Students Diagnosed with ADHD, Objective Measurement in a Natural Learning Environment

**DOI:** 10.3390/ijerph18083870

**Published:** 2021-04-07

**Authors:** Mireia Sempere-Tortosa, Francisco Fernández-Carrasco, Ignasi Navarro-Soria, Carlos Rizo-Maestre

**Affiliations:** 1 Department of Computer Science and Artificial Intelligence, University of Alicante, 03690 Alicante, Spain; mireia@ua.es; 2 Development Psychology and Teaching Department, University of Alicante, 03690 Alicante, Spain; ignasi.navarro@ua.es; 3 Department of Architectural Constructions, University of Alicante, 03690 Alicante, Spain; carlosrm@ua.es

**Keywords:** assessment, diagnosis, ADHD, motion capture, hyperactivity, teaching innovation, classroom

## Abstract

Attention deficit hyperactivity disorder is the most common neuropsychological disorder in childhood and adolescence, affecting the basic psychological processes involved in learning, social adaptation and affective adjustment. From previous research, the disorder is linked to problems in different areas of development, with deficiencies in psychological processes leading to the development of the most common characteristics of the disorder such as inattention, excess of activity and lack of inhibitory control. As for the diagnosis, in spite of being a very frequent disorder, there are multiple controversies about which tools are the most suitable for evaluation. One of the most widespread tools in the professional field is behavior inventories such as the Strengths and Difficulties Questionnaires for Parents and Teachers or the ADHD Rating Scale-V. The main disadvantage of these assessment tools is that they do not provide an objective observation. For this reason, there are different studies focused on recording objective measures of the subjects’ movement, since hyperkinesia is one of the most characteristic symptoms of this disorder. In this sense, we have developed an application that, using a Kinect device, is capable of measuring the movement of the different parts of the body of up to six subjects in the classroom, being a natural context for the student. The main objective of this work is twofold, on the one hand, to investigate whether there are correlations between excessive movement and high scores in the inventories for the diagnosis of ADHD, Rating Scale-V and Strengths and Difficulties Questionnaire (SDQ) and, on the other hand, to determine which sections of the body present the most significant mobility in subjects diagnosed with ADHD. Results show that the control group, composed of neurotypical subjects, presents less kinaesthetic activity than the clinical group diagnosed with ADHD. This indicates that the experimental group presents one of the main characteristics of the disorder. In addition, results also show that practically all the measured body parts present significant differences, being higher in the clinical group, highlighting the head as the joint with the highest effect size.

## 1. Introduction

Attention Deficit Hyperactivity Disorder (ADHD) is the most common neuropsychological disorder in childhood and adolescence [1,2,3]. It is a chronic and persistent neurodevelopmental disorder, starting in early childhood, that affects the basic psychological processes involved in learning, social adaptation and affective adjustment [4]. ADHD affects 3–7% of children, being more frequent among boys than girls. A diagnosis is usually made between 5 and 8 years of age, coinciding with the beginning of school [5]. Depending on its manifestations, three diagnostic categories can be found: ADHD hyperactive/impulsive type, ADHD inattentive type and ADHD combined presentation (when it meets the criteria for both types); these last two are usually seen mainly in clinical practice [6].

The diagnosis is mainly based on behavioral symptoms and is made before the age of 12. For a diagnosis to be made, the child must present alterations in at least two environments such as at home, social situations and/or school. Furthermore, these changes cannot be due to the existence of another mental disorder. Cases are usually detected in the school environment due to the learning difficulties that these children present and which often lead to school maladjustment. In addition to academic performance, areas such as emotional adjustment and peer relationships are also affected at this age. If it persists into adulthood, it can lead to low socioeconomic status, difficulties and changes in work and other problems in daily life, especially if the individual does not receive treatment [7].

When regarding the etiology of ADHD, it cannot be assessed from a singular perspective, with scientific literature collecting information from various fields to try to give an explanation to this disorder. From a genetic point of view, the high heritability of the disorder, modulated by the interaction with the environment, has been demonstrated [8,9]. According to the anatomical level, differences have been found at the structural level of the brain [10,11,12] and one of the most supported theories is neurochemistry, which defends that the disorder is due to an alteration in neurotransmission, a dysfunction in the circuits mediated by dopamine and noradrenaline [13,14]. It is this variance in the system that produces alterations in psychological processes.

From the follow-up of the research developed on the basis of this last theory, it has been observed that the disorder is linked to problems in different areas of cognitive development, such as working memory [15], processing speed [16,17,18], general IQ [7,19] and psychomotor coordination speed [20]. Derived from the deficiencies in these psychological processes appear the most significant characteristics of ADHD such as inattention, over-activity and lack of inhibitory control [21], as well as a marked difficulty in the psychological processes involved in executive functions [4,22,23,24,25,26]. These cognitive skills were described by Barkley [27], as those self-directed actions that the subject uses to self-regulate [28]. In other words, they are a set of cognitive skills comprised in a complex multimodal system, formed by different elements that interact to achieve problem solving and goal-directed behavior, receiving the support of the previously mentioned cognitive components to achieve these goals.

Based on this previous knowledge, there have been many efforts to try to operationalize, measure and detect deficiencies in the cognitive processes and functions of the executive system involved in ADHD. However, controversy remains about what these neuropsychological variables are and which tools are the most suitable for their evaluation [29].

One of the most widespread tools in the professional field dedicated to diagnosing and intervening with subjects who have ADHD are behavioral inventories. This tool, through a series of questions answered mostly by family and teachers, determines whether the behaviors related to the disorder are present to a sufficient degree to carry out a diagnosis. The most widespread inventories are the Strengths and Difficulties Questionnaires for Parents and Teachers [30], the ADHD Rating Scale-V [31] or the Conners’ Rating Scale for Parents and Teachers [32]. However, these instruments are highly subject to biases such as the knowledge regarding ADHD that the person responding has or their experience with the subject that the questions are about. For example, the Strengths and Difficulties Questionnaire (SDQ) has a scale made up of 25 Likert-type items of three points. Among the items it is possible to find: Constantly fidgeting or squirming. The ADHD Rating Scale-V presents a scale made up of 18 Likert-type items of four points and the Conners’ Rating Scale, also of the Likert type, both present an item such as: Fidgets with hands or feet or squirms in seat. These items represent ADHD symptoms according to criteria established in the DSM-5 such as “Fidgeting with or tapping hands or feet, squirming in seat”. These ratings can be very subjective, as each person completing the questionnaire must choose between it being “just a little true” or “pretty much true”.

Therefore, the main disadvantage that we find in these evaluation systems is that they do not provide an objective observation. Thus, the imprecision of the diagnosis of ADHD based on subjective criteria [33], coupled with the fact that hyperactivity is one of the core symptoms of this disorder [34,35] have led to that for more than a decade, studies have been conducted to record objective measures of movement in subjects [34,36,37,38,39,40,41]. However, these studies have a number of limitations. Firstly, some use accelerometers (actigraphy and inertial measurement units), where a device has to be placed in the body of the subjects, thus limiting its ecological validity and, secondly, studies that use infrared only have the capacity of analyzing a certain part of the body. Likewise, the Kinect device has also been used for movement measurements in subjects diagnosed with ADHD but in a non-ecological environment and outside the classroom [39,42].

Trying to solve these mentioned limitations, the ADHD Movements application has been programmed. This application uses a Kinect device to objectively measure the movement of the subjects without the use of any device on the body. Furthermore, as it is a small device, it can be introduced into the natural environment without being invasive. ADHD Movements detects the visible skeletons and their body sections. The application then draws each of the joints on the screen and records, analyzes and counts the amount of movement and distance traveled by each joint of each subject. More detailed information on the development of this application can be found at [43]. In this way, this application tries to provide objectivity to movement measurements such as “Fidgeting with or tapping hands or feet and squirming in seat”, indicated in the DSM-5, while allowing collecting information about motor behavior in a student’s natural context.

The main objective of this research is to investigate whether there are correlations between excessive movement and high scores in the inventories for the diagnosis of ADHD, Rating Scale-V and SDQ. In turn, we try to determine which sections of the body present the most significant mobility in those subjects diagnosed with ADHD. To carry out this study, a series of classroom sessions were held. These sessions consisted of a study techniques workshop with boys and girls, where the movement of the different parts of the body was measured during the workshop, using the developed application.

## 2. Method

### 2.1. Procedure

Firstly, the project was presented to the Clinical Research Ethics Committee of the General Hospital of Alicante (CEIC-HGUA) for evaluation. After the evaluation, approval was obtained which allowed the selection of the children.

The sample of the experimental group was obtained from the Children’s Mental Health Unit (USMI) of the General Hospital of Alicante, taking into account children between 7 and 12 years of age diagnosed with ADHD. The procedure was then explained to the parents and guardians of the children so that they could accept the conditions of the study. Once the legal permits were obtained, several dates of availability for a workshop of study techniques were proposed to each of the families involved.

In parallel to the main study, a procedure was carried out to form a sample for a control group. For this purpose, we contacted the public school CEIP Azorín in the town of San Vicente del Raspeig. The sample of the group was made with the same characteristics as the experimental group, in terms of age and gender.

Finally, groups of mixed students were created, with a homogeneous number of subjects from the clinical and control groups. All the workshops were given by two speakers, for approximately 45 min, where different cognitive tools were explained to facilitate the learning process. The speakers did not intervene if during the session there was an episode of absent-mindedness on the part of a subject, since the objective was to take movement measurements. Additionally, prior to data collection, the inventories for the diagnosis of ADHD, Rating Scale-V and SDQ, were applied to all participants, in order to verify the presence of symptoms and the degree of appearance in all components of the sample.

### 2.2. Participants

The sample was composed of 65 children, aged between 7 and 12 years. The children were classified in two groups: Experimental group and control group. The experimental group was composed of 32 children with a clinical diagnosis of ADHD: 24 boys and 8 girls. In turn, of the 32 children, 24 had a diagnosis of ADHD with combined presentation and 8 predominantly inattentive presentation, according to the criteria of the DSM-5 [6]. The age range of the participants in the experimental group was between 7 and 12 years (Mean=9.75 and StandardDeviation=1.50). Of the total number of subjects in the experimental group, 23 (71.9%) were prescribed regular pharmacological treatment to alleviate the symptoms of the disorder. Of these 23 subjects taking treatment, 16 (69.6%) are prescribed methypphenidate, 6 (26.1%) amphetamine and 1 (4.4%) atomoxetine. The control group was composed of 33 children from a neurotypical sample: 17 boys and 16 girls. The age range of the participants in the control group was between 8 and 12 years (M=9.69 and SD=1.50).

### 2.3. Instruments

- ADHD Rating Scale V (ADHD RS-V) [44,45]. Scale made up of 18 items with a 4-point Likert type scale (0 = never or rarely; 1 = sometimes; 2 = frequently; 3 = very frequently). Each item represents the symptoms of ADHD according to the criteria of the DSM-5 [6]. It has two subscales: Inattention and hyperactivity. It has a version for teachers and another for parents, which meets the criteria of “presence of the symptomatology in at least two environments”. The evaluator’s response is centered on the frequency of the evaluated subject’s behavior in the last six months. Furthermore, from both a psychometric and applied point of view, the ADHD RS-V has many strengths: It is normalized by age, gender and type of assessor [44], it has good results in terms of reliability, internal consistency and validity, it has demonstrated clinical utility for the diagnosis of ADHD [46,47] and has been successfully used in studies examining the efficacy of drug treatments [48,49].
- Strengths and Difficulties Questionnaire (SDQ) [30,50]. The instrument evaluates different emotional and behavioral problems in children and adolescents between 4 and 16 years of age. There are two versions of the instrument in Spanish: For parents and for teachers. It is composed of 25 items with a 3-point Likert response scale (0 = not true; 1 = somewhat true; 2 = totally true). In addition, the extended version (used in this study) has an “impact supplement”, which assesses the degree to which the child’s difficulties interfere with his/her daily life. The items are grouped into 5 scales: Emotional symptoms, behavioral problems, hyperactivity/inattention, peer relationship problems, and prosocialbehavior. The reliability of this instrument through Cronbach’s α in a Spanish sample is 0.77 [51].
- Kinect device. The Kinect device was originally developed for Microsoft’s XBox360 video game console to provide a natural interface that allows interaction using body movement, gestures and voice commands so that users can interact with the console without the need for physical contact with a video game controller. However, due to the characteristics of the device and the possibility of developing their own applications using different libraries (both free and Microsoft’s own) its use has not been limited only to video games. In fact, it has been considered an innovative interface of interaction between people and computers, besides highlighting its simplicity in data acquisition [52]. Kinect is a horizontal device with a camera, infrared emitter, depth sensors and a set of microphones embedded in a rectangular box. Using depth and vision data, based on automatic learning algorithms, the Kinect device detects the human body. In this sense, Kinect detects the different parts of the body and represents them by means of a skeleton formed by 25 joints. Each joint is identified by its name and its position is measured with three coordinates (x,y,z), with (x,y) indicating the position, while (*z*) represents the distance to the sensor. From these coordinates it is possible to perform the necessary calculations to detect a movement, movement patterns, gestures, postures, etc. Based on this device, ADHD Movements application has been used. It allows us to know the amount of movement of each joint of all the subjects during each minute of the study techniques workshop.

### 2.4. Media Architecture

**Figure 1:**
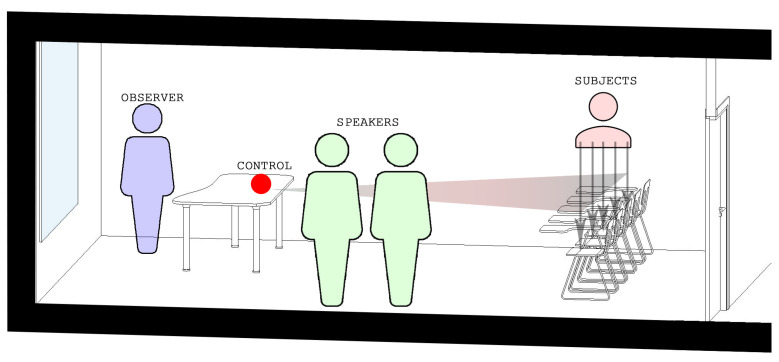
Image showing the arrangement in the classroom of the different elements used in the study.

For the practical elaboration of the project, we looked for a unique space with specific conditions where the activities could be carried out for all the assistants. The chosen space was a classroom at the University of Alicante that had chairs with a built-in table as it would facilitate the use of the Kinect device since this type of chairs allow a view of the whole body (Figure 1).

The different activities carried out in the workshop sessions were organized by age groups, combining 50% of the children from the experimental and control groups. One of the limitations of the Kinect device is the limitation of 6 bodies, therefore, each of the sessions was programmed with a maximum of 6 children and a minimum of 4.

Children from the experimental group who were taking some kind of medication were invited to attend a new workshop to “expand and review” the previously explained study techniques. For this particular session, they had to attend without taking the prescribed medication during the previous days to alleviate the symptoms derived from the disorder.

The predisposition of all the participating families is to be thanked, since they agreed to all requests and petitions. Therefore, the children in the experimental group were studied on two occasions, that is, two movement measurements were taken; one with the medication and the other without. In order to ensure the capture of the movement of the subjects, and to prevent any type of technological incident, two Kinect devices were used in each of the workshops.

## 3. Data Analysis

To carry out the statistical analysis, a quantitative methodology was applied in which descriptive and difference analyses were carried out. Firstly, the frequencies and central tendency measures were calculated. Furthermore, prior to the analysis of differences and in order to choose the most suitable hypothesis test, the assumption homoscedasticity (Levene test) was checked [53]. Then, as data follow a normal distribution, the Student’s *t*-test was used and the magnitude of the effect sizes of the differences found were tested with a *d* index (standardized mean differences): Small (0.20≤d≤0.50), moderate (0.50≤d≤0.79), and large (d≥0.80) [54]. All data were analyzed by a member of the team with experience and training in methodology, using SPSS Statistical software package, IBM SPSS Statistics v25.

## 4. Results

Following the objectives established, the results can be found below. Firstly, the differences in means in the hyperactivity subscale of the ADHD RS-V questionnaire are presented, followed by the results obtained for the hyperactivity/inattention subscale of the SDQ questionnaire. Finally, the differences in objective movement recorded by the Kinect device are analyzed from two different perspectives: Experimental group vs. control group, and experimental group with and without taking their usual medication.

### 4.1. Differences in the Hyperactivity Subscale Score According to the ADHD RS-V Questionnaire

Table 1 offers the means and statistically significant differences found in the hyperactivity subscale of the ADHD RS-V questionnaire (teachers version and parents version), between the experimental group and the control group.

The results indicate that, after the tutors and parents answers, the subjects of the experimental group present a higher mean score than those of the control group for the hyperactivity subscale.

Furthermore, following the criteria proposed by Cohen [54], the magnitude of the difference is high both for the questionnaires completed by the teachers (d=1.57), as those completed by the parents (d=0.83).

### 4.2. Differences in Hyperactivity Subscale Score According to the SDQ Questionnaire

Table 2 offers the means and statistically significant differences found in the hyperactivity subscale of the SDQ questionnaire (teachers version and parents version), between the experimental group and the control group.

After the analysis of the tutors’ and parents’ answers, the results indicate that the subjects with a diagnosis of ADHD obtain higher average scores on the hyperactivity subscale, than the subjects of the control group.

Furthermore, following the criteria proposed by Cohen [54], the magnitude of the difference is high if we consider the questionnaires filled out by teachers (d=1.57) and high in the case of those filled out by parents (d=0.83).

### 4.3. Study of Mean Differences for Register of Movement of the Joints Measured by Kinect for the Clinical and Control Groups

Firstly, Table 3 shows the mean scores and statistically significant differences found between the clinical group and the control group, for the different joints measured. The data shows that, except for the left ankle, the clinical group have higher movement scores in all of the measured body sections, when compared to the scores of the control group. The differences found between the clinical and control groups are significant for 14 of the 17 joints measured. Furthermore, the magnitudes of the differences found between the joints are also calculated. In the case of the middle spine, neck, head, left shoulder, left elbow, left wrist, right shoulder, right elbow, right knee and upper spine these magnitudes are small and range from 0.21 to 0.46. In turn, the joints of the base of the spine, right wrist, left hip, left knee and right hip also scored statistically significant differences; however, the magnitudes found do not reach the values determined by Cohen [54] to be considered relevant scores. Regarding the left ankle, contrary to what has been observed so far, the control group obtained a higher mean score than the clinical group, although this difference was not statistically significant.

### 4.4. Study of Mean Differences for the Recording of Joint Movement Measured by Kinect, for the Clinical Group with and without Taking Their Usual Medication

**Figure 2:**
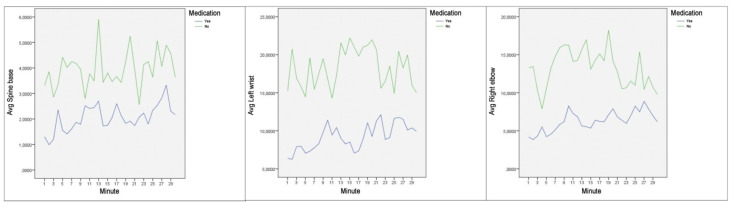
Average movement per minute for spine base (**left**), left wrist (**center**) and right elbow (**right**).

Hereafter, we present the mean scores and the differences of the clinical group in relation to joint movement at two different times, having taken the usual prescribed medication and, the same subjects, without taking the medication that palliates the symptoms of an attention deficit disorder (Table 4 and Figure 2). The data obtained show that there are statistically significant differences for all the items analyzed, with the highest mean differences when the subjects diagnosed with ADHD had not taken the prescribed medication. As in the previous statistics, the magnitude of the differences was calculated, resulting in a small magnitude for the joints of the middle spine, neck, left shoulder, right shoulder and upper spine; the scores were between 0.22 and 0.42. In turn, the magnitude of the differences is moderate for the joints of the left elbow, left hip, left knee, left ankle, right knee and right ankle; ranging from 0.56 to 0.79. Finally, the magnitude of the differences is high for the base of the spine, left wrist, right elbow, right wrist and right hip; ranging from 0.83 to 1.12. Regarding the head, although the difference found in both records of the clinical group is statistically significant, the scores obtained do not reach the values determined by Cohen [54] to be considered relevant.

## 5. Discussion of the Results

Below, the results obtained in this study will be discussed, both those most significant and related to our objectives, as well as those less significant and that do not align with the aims of the present study. In order to justify our objectives, the statistical analysis of the collected data was carried out and the results are presented hereafter. Firstly, it is important to highlight the result of the comparison of means between the control group and the experimental group on the hyperactivity subscale of the ADHD RS-V questionnaire and the hyperactivity subscale of the SDQ questionnaire, which detect differences of a high magnitude both in the school context (d=1.57) and in the family context (d=0.83), showing that the control group, composed of neurotypical subjects, presents a lower kinaesthetic activity than the clinical group diagnosed with ADHD. This reveals that the experimental group presents one of the main characteristics of the disorder [34,35]. On the other hand, when we compare the differences between the mean scores of the clinical group and the control group it can be observed that practically all the measured body parts present significant differences, with higher scores in the case of the clinical group.

Of these differences, it is relevant to highlight the head as the joint with the highest size effect (d=0.46), since this body part is classed as dominant when assessing what the individual fixes his/her attention on, as well as the quality of attention paid to stimuli [36,55,56]. In turn, if we observe the following highest scores, we find the neck (d=0.36) and the left (d=0.32) and right (d=0.39) shoulders, components of the human body that accompany, at the motor level, the turning of the head when the subject changes his/her attentional focus to a different stimulus. Therefore, the application developed is considered valid in the comparison of means, when we compare individuals with and without ADHD who pay attention to the same attentional stimulus.

Finally, in line with a recent study investigating the effect of methylphenidate in children with ADHD [57], which found that head movements were not significantly different between medicated and non-medicated ADHD groups, our study corroborates differences in the head, neck, left shoulder and right shoulder, when comparing the clinical group under the effects of medication and the same group of non-medicated subjects. However, the size of effect is one of the smallest that we find in relation to other joints measured between these two groups, even in the case of the head, the difference is the smallest of all measures carried out.

This leads us to conclude that, for our sample, there are significant differences in the mean difference for the 17 body parts studied between the control group and the clinical group. These differences are greater in the sections of the body that are more related to the focus of attention on a learning stimulus. In turn, when we compared the frequency of movements of the different body sections in the clinical group with and without medication, we detected that the smallest differences were in the sections that we had previously found wider discrepancies between two groups. This fact leads us to ask ourselves if medication has a lesser effect when controlling excessive movement in the segments of the body that present a greater relationship with attention, which would lead us to understand that psychostimulants improve general body control, but may not be as effective in executive functions, the cognitive substratum of attention control.

## 6. Limitations and Future Lines of Research

In view of the results obtained in this research, we consider essential that, through future lines of research, this pilot study be continued by validating the results. Firstly, it would be interesting to focus the effort of mediation and data collection on those anatomical components that can provide us with more information about the ability to focus attention on an auditory or visual stimulus, to be more rigorous in the conception of movement of these components and even to assess different types of movement, linking them either to those derived from the kinesthesia that accompanies the rest of the anatomical components of the subject’s body or to movements that are directly related to the defocusing of attention on the learning stimuli.

For this, it will be necessary to overcome the main limitations of the current study, mainly the small number of subjects that make up both the clinical and control groups. While the results obtained are significant, it is difficult to assess them as generalizable to the entire clinical population diagnosed with ADHD. For this tool to become a valid instrument to support the diagnosis, it is important that it has a scale composed of neurotypical population and clinical population, which would allow professionals who use it in educational contexts, to support their observation of data and allow them to guide a diagnosis. Therefore, through future work, we consider essential to collect a sample that will provide more solidity and transference to the obtained results.

In addition, we also found as a limitation, having measured a high number of sections of the body of the research subjects, many of these anatomical sections being of little relevance. It would be convenient to focus on those anatomical sections that show significant differences between clinical and control groups. Therefore it would be highly interesting to study the correlation between the control of movement/orientation of the head, neck and right/left shoulder sections of the body, the taking or not of the medication prescribed to alleviate the symptoms of ADHD and the effectiveness in performing tasks that require executive functions.

Finally, after collecting a larger sample of subjects, it would be very interesting to determine two experimental groups, one composed of students diagnosed with ADHD with combined predominance and the other with students diagnosed with ADHD with inattentive predominance. From what we have observed in the practical application of the measuring instrument, it is very likely that kinaesthetic patterns could be determined for each diagnostic presentation.

## Figures and Tables

**Table 1:** Differences in Hyperactivity of the Attention Deficit Hyperactivity Disorder (ADHD) Rating Scale V (RS-V), teachers version and parents version.

Variable Group	Levene Test		Experimental Group		Control Group		Statistical Significance and Magnitude of Differences			
	***F***	***p***	***M***	***SD***	***M***	***SD***	***t***	***d.f.***	***p***	***d***
Hyperactivity Tutors	1.662	0.202	7.33	2.08	3.48	2.77	6.17	61	<0.001	1.57
Hyperactivity Parents	0.051	0.822	7.06	2.31	5.15	2.27	3.35	63	0.001	0.83

**Table 2:** Score differences for the hyperactivity subscale of the Strengths and Difficulties Questionnaire (SDQ) (teachers and parents version) between the control group and experimental group.

Variable Group	Levene Test		Experimental Group		Control Group		Statistical Significance and Magnitude of Differences			
	***F***	***p***	***M***	***SD***	***M***	***SD***	***t***	***d.f.***	***p***	***d***
Hyperactivity Tutors	1.66	0.20	9.33	2.09	5.48	2.77	6.17	61	<0.001	1.57
Hyperactivity Parents	0.05	0.822	9.06	2.31	7.15	2.27	3.36	63	0.001	0.83

**Table 3:** Differences in means of movement of the different joints recorded by Kinect between the experimental group and the control group.

Kinect Joint	Levene Test		Experimental Group		Control Group		Statistical Significance and Magnitude Differences			
	F	*p*	M	SD	M	SD	*t*	d.f.	*p*	*d*
Spine base	45.42	<0.001	2.92	2.41	2.53	1.82	4.73	2512.24	<0.001	0.18
Spine mid	74.18	<0.001	2.14	1.76	1.62	1.05	9.59	2665.48	<0.001	0.34
Neck	64.04	<0.001	3.01	2.36	2.26	1.60	9.70	2612.67	<0.001	0.36
Head	102.41	<0.001	3.48	2.52	2.44	1.67	12.79	2628.94	<0.001	0.46
Left shoulder	46.76	<0.001	2.76	2.18	2.11	1.66	8.62	2496.40	<0.001	0.32
Left elbow	46.53	<0.001	7.72	6.06	6.54	4.79	5.50	2448.92	<0.001	0.21
Left wrist	53.83	<0.001	13.41	10.31	12.84	7.87	1.62	2497.42	0.105	-
Right shoulder	111.21	<0.001	2.76	2.25	2.00	1.33	10.97	2665.93	<0.001	0.39
Right elbow	15.46	<0.001	9.50	7.85	7.92	7.00	5.38	2265.50	<0.001	0.21
Right wrist	92.18	<0.001	16.79	14.08	15.38	10.05	3.00	2571.63	0.003	0.11
Left hip	27.87	<0.001	3.02	2.35	2.72	1.93	3.57	2393.97	<0.001	0.14
Left knee	4.35	0.037	3.95	4.00	3.21	4.49	4.26	1890.72	<0.001	0.18
Left ankle	10.13	0.001	8.16	7.72	8.23	8.70	−0.205	1883.48	0.838	-
Right hip	25.15	<0.001	3.02	2.34	2.72	1.97	3.47	2356.94	0.001	0.14
Right knee	24.75	<0.001	4.23	4.79	3.14	3.47	6.76	2554.91	<0.001	0.25
Right ankle	0.635	0.426	8.95	8.54	8.56	9.54	1.08	2666	0.279	-
Spine shoulder	73.26	<0.001	2.65	2.21	1.97	1.40	9.61	2650.59	<0.001	0.35

**Table 4:** Differences in means of movement of the different joints recorded by Kinect between the experimental group with and without taking their usual medication.

Kinect Joint	Levene Test		Experimental Group (with Medication)		Experimental Group (without Medication)		Statistical Significance and Magnitude Differences			
	**F**	***p***	**M**	**SD**	**M**	**SD**	***t***	**d.f.**	***p***	***d***
Spine base	32.30	<0.001	2.05	1.83	3.94	2.60	16.92	1370.59	<0.001	0.83
Spine mid	0.001	0.974	1.80	1.57	2.53	1.89	8.61	1676	<0.001	0.42
Neck	<0.001	0.996	2.75	2.18	3.32	2.51	4.93	1676	<0.001	0.24
Head	4.63	0.031	3.31	2.45	3.68	2.59	3.02	1609.82	0.002	0.15
Left shoulder	0.381	0.537	2.48	2.04	3.08	2.29	5.69	1676	<0.001	0.28
Left elbow	26.33	<0.001	5.81	5.23	9.94	6.20	14.61	1526.61	<0.001	0.72
Left wrist	15.68	<0.001	9.21	8.60	18.28	9.99	19.75	1543.72	<0.001	0.97
Right shoulder	0.490	0.484	2.43	2.04	3.15	2.41	6.60	1676	<0.001	0.32
Right elbow	186.14	<0.001	6.28	4.87	13.23	8.91	19.39	1163.32	<0.001	0.95
Right wrist	147.05	<0.001	10.24	9.40	24.37	14.77	22.94	1281.24	<0.001	1.12
Left hip	13.56	<0.001	2.22	1.96	3.94	2.43	15.70	1492.09	<0.001	0.77
Left knee	102.38	<0.001	2.91	2.91	5.15	4.71	11.50	1256.51	<0.001	0.56
Left ankle	276.86	<0.001	5.71	4.61	11.01	9.44	14.25	1090.70	<0.001	0.70
Right hip	11.56	0.001	2.17	1.94	4.00	2.39	16.95	1494.15	<0.001	0.83
Right knee	212.30	<0.001	2.90	2.80	5.76	6.00	12.13	1064.31	<0.001	0.60
Right ankle	266.49	<0.001	5.93	5.37	12.43	10.07	16.12	1146.82	<0.001	0.79
Spine shoulder	0.074	0.786	2.42	1.99	2.91	2.41	4.49	1676	<0.001	0.22
